# Clinical Characteristics, Outcomes, and Risk Factors of Disease Severity in Patients With COVID-19 and With a History of Cerebrovascular Disease in Wuhan, China: A Retrospective Study

**DOI:** 10.3389/fneur.2021.706478

**Published:** 2022-01-11

**Authors:** Mengzhen Li, Zehui He, Jiecong Yang, Qihua Guo, Heng Weng, Jielian Luo, Baoying Gong, Wanzhen Cui, Banghan Ding, Jianwen Guo

**Affiliations:** ^1^The Second School of Clinical Medicine, Guangzhou University of Chinese Medicine, Guangzhou, China; ^2^Department of Clinical Epidemiology, The Second Affiliated Hospital of Guangzhou University of Chinese Medicine, Guangzhou, China; ^3^Department of Big Medical Data, Health Construction Administration Center, The Second Affiliated Hospital of Guangzhou University of Chinese Medicine, Guangzhou, China; ^4^Department of Emergency, The Second Affiliated Hospital of Guangzhou University of Chinese Medicine, Guangzhou, China; ^5^Department of Neurology, The Second Affiliated Hospital of Guangzhou University of Chinese Medicine, Guangzhou, China; ^6^State Key Laboratory of Dampness Syndrome of Chinese Medicine, The Second Affiliated Hospital of Guangzhou University of Chinese Medicine, Guangzhou, China

**Keywords:** COVID-19, cerebrovascular disease, clinical characteristics, adverse outcomes, risk factors

## Abstract

**Background and Purpose:** Coronavirus disease 2019 (COVID-19) rapidly resulted in a pandemic. Information on patients with a history of cerebrovascular disease (CVD) infected with severe acute respiratory syndrome coronavirus 2 (SARS-COV-2) is limited. This study investigated the clinical features and the risk factors of developing adverse outcomes in patients with COVID-19 and with previous CVD.

**Methods:** This was a single-center retrospective clinical study including all the confirmed cases of COVID-19 at Wuhan Huoshenshan Hospital from February 4 to April 7, 2020. Differences in clinical characteristics were compared between patients with and without a history of CVD. The incidences of severe events comprising all-cause death, intensive care unit admission, shock, and mechanical ventilation usage during hospitalization in two groups were compared using propensity score matching analysis and multivariate logistic regression analyses. Besides, the risk factors of developing severe events in patients with COVID-19 who also have history of CVD were analyzed.

**Results:** A total of 2,554 consecutive patients were included in our study, of whom 109 (4.27%) had a medical history of CVD. Patients with CVD tend to be older and with more comorbidities, including hypertension, diabetes, coronary heart disease, and chronic obstructive pulmonary disease. The levels of white blood cell, neutrophil, C-reactive protein, creatine kinase isoenzymes, and lactate dehydrogenase were higher, whereas the levels of lymphocyte and albumin were lower in the CVD group. Compared to those without CVD, patients with CVD were more likely to have severe events after age matching (12.8 vs. 5.7%, *P* = 0.012). After adjusting for the confounding effects of age, sex, smoking, and comorbidities, the odds ratio for developing severe events with a history of CVD was 2.326 (95% CI, 1.168–4.630; *P* = 0.016). Besides, patients with CVD, either with decreased lymphocyte count (OR 9.192, 95% CI, 1.410–59.902, *P* = 0.020) or increased blood urea nitrogen (OR 5.916, 95% CI, 1.072–32.641, *P* = 0.041), had a higher risk of developing severe events during hospitalization.

**Conclusions:** Patients with CVD history tend to have adverse clinical outcomes after being infected with SARS-COV-2. Decreased lymphocyte counts and increased blood urea nitrogen levels may be risk factors for adverse outcomes in patients with COVID-19, and had CVD.

## Introduction

Coronavirus Disease 2019 (COVID-19) has now developed into a global pandemic with high morbidity and mortality. As per the WHO situation report of December 20, 2020, more than 60 million cases have been confirmed worldwide (1). A model of SARS-COV-2 transmission projected that SARS-COV-2 could enter a long-term circulation (2). Thus, with the situation of relative shortage of medical resources, early recognition of clinical predictors associated with COVID-19 deterioration, and improvement of the management of the high-risk population may be the effective methods to judicious use of limited medical resources and to reduce the mortality rate (3). Current studies have demonstrated a close relationship between COVID-19 severity and underlying comorbidities, such as chronic obstructive pulmonary disease (COPD), hypertension, and diabetes (4, 5). However, the clinical features of patients with COVID-19, who also have chronic neurological conditions, have not been fully understood. Here, we compared the difference in clinical characteristics and outcomes between patients with and without a history of cerebrovascular disease (CVD) in a COVID-19 cohort of 2,554 hospitalized cases, and explored if the history of CVD was associated with an increased risk of critical conditions. Besides, clinical risk factors for developing severe events in patients, who have both COVID-19 and CVD history, were analyzed.

## Materials and Methods

### Experimental Design and Participants Enrolled

This was a single-center retrospective clinical study, including 2,554 confirmed patients with COVID-19 at Wuhan Huoshenshan Hospital from February 4 to April 7, 2020. The analysis included all the hospitalized patients with COVID-19, except for the 18 participants with no complete electronic medical records. Confirmed COVID-19 cases were defined as tested positive for SARS-COV-2 by real-time reverse-transcriptase PCR (RT-PCR) assay for nasal or pharyngeal swab specimens. This study was approved by the Ethics Committee of Guangdong Provincial Hospital of Traditional Chinese Medicine (No. ZE2020-049-01). The committee waived the need of written informed consent due to the retrospective nature of this study.

### Data Acquisition

The data including demographic information, medical history, therapeutic regimens, laboratory findings, and clinical outcomes were obtained from electronic medical records. With reference to *The Guidelines on 2019-nCoV Treatment and Prevention issued by the NHC* in February 2020, those meeting even one of the following conditions were diagnosed as critical type: (1) Respiratory failure was observed, and mechanical ventilation was required; (2) Shock; and (3) Organ failure requiring intensive care unit (ICU) monitoring and treatment. Progressions to critical type and to an all-cause death were defined as the severe events in this study. Following discharged standards in the *Guidelines* (mentioned above), those who meet all of the following conditions can be discharged from the hospital: (1) Body temperature returned to normal for more than 3 days; (2) Respiratory symptoms were obviously improved; (3) Pulmonary imaging showed that acute exudative lesions were significantly improved; and (4) Nucleic acid tests for respiratory tract specimens such as sputum and nasopharyngeal swabs are negative twice in a row (sampling time interval at least 24 h).

### Statistical Analysis

Data were conducted to descriptive analysis. Continuous variables with normal distribution were described as the mean (standard deviation). Continuous variables with non-normal distribution were described as the median (interquartile range). The *T*-test and Mann–Whitney *U*-test were used to perform inter-group comparisons of continuous variables, between normally distributed and abnormally distributed. Categorical variables were described as the frequency (percentage). Differences in categorical variables were analyzed with the chi-square test or Fisher's exact test. The propensity score of 1:5 matching (PSM) analysis based on different age-level ( ≤ 29, 30–39, 40–49, 50–59, 60–69, 70–79, and 80–89, ≥90), between patients without and patients with CVD, was conducted by “MatchIt” package in R software (version 4.1.1) using the “nearest matching” method (6). The incidences of severe events were compared in age-matched groups. Logistic regression models were used to analyze the relationship between the history of CVD and the presence of severe events, adjusting for the confounding effect of age, smoking, and having multiple medical comorbidities. Then, we further explored the risk factors for developing severe events in patients who had CVD. One hundred nine patients with a history of CVD were divided into two groups based on the presence of severe events. The relationships between the incidence of severe events and variables including baseline characteristics (age, sex, smoking history, and multiple medical comorbidities), laboratory examinations, time from symptoms to admission, and drug treatment were analyzed in univariate regression analysis. Next, variables with statistical significance (*P* < 0.05) in univariate analysis were included in multivariate regression analysis. No data imputation was made for missing values. Statistical analysis was performed using SPSS 22.0 software.

## Results

### Baseline Characteristics of Patients With COVID-19, With or Without CVD History

Out of 2,554 enrolled cases, 109 (4.28%) had a medical history of CVD. Compared to patients without CVD, patients with CVD tend to be older [71.0 (interquartile range IQR:15) vs. 59 (IQR:18) *P* < 0.001], and a higher proportion of these patients had comorbidities of hypertension (74.3 vs. 29.3%, *P* < 0.001), diabetes (32.1 vs. 14.15%, *P* < 0.001), coronary heart disease (24.8 vs. 6.4%, *P* < 0.001), or chronic obstructive pulmonary disease (COPD) (4.6 vs. 0.8%, *P* = 0.001; Table 1).

**Table 1:** Baseline characteristics of coronavirus disease 2019 (COVID-19) patients with or without history of cerebrovascular disease (CVD).

	**History of CVD *n* = 109**	**No History of CVD *n* = 2,445**	***P*-Value**
Gender (Female) (proportion)	61 (56.0)	1,231 (50.3)	0.251
Age (year), median (IQR)	71.0 (15.0)	59.0 (18.0)	<0.001
smoking (proportion)	12 (11.0)	185 (7.6)	0.187
**Comorbidities (proportion)**			
Hypertension	81 (74.3)	713 (29.2)	<0.001
Diabetes	35 (32.1)	355 (14.5)	<0.001
Coronary heart disease	27 (24.8)	156 (6.4)	<0.001
Atrial fibrillation	1 (0.9)	32 (1.3)	1.000
COPD	5 (4.6)	20 (0.8)	0.001
Chronic liver diseases	8 (7.3)	142 (5.8)	0.506
Chronic kidney diseases	6 (5.5)	59 (2.4)	0.090
Malignant tumors	2 (1.8)	52 (2.1)	1.000
Hematological diseases	1 (0.9)	32 (1.3)	1.000

*Data are described as median (IQR) or proportion (%); χ^2^ test or T-test was used to calculate P-value when appropriate. IOR indicates interquartile range; COPD indicates chronic obstructive pulmonary disease*.

### Clinical Characteristics and Laboratory Findings of Patients With COVID-19, With or Without CVD History

Patients with a history of CVD had more severe clinical conditions compared to patients without CVD. The percentages of severe (45.0 vs. 27.0%) and critical cases (3.7 vs. 1.1%) (*P* < 0.001) were much higher in the CVD group at the time of the hospital admission. Furthermore, there were also significant differences in laboratory examinations between the two groups. As shown in Table 2, white blood cell count [5.80 10^9^/L (IQR: 5.85) vs. 5.40 10^9^/L (IQR: 2.3), *P* = 0.001], neutrophil count [3.37 10^9^/L (IQR: 3.02) vs. 3.13 10^9^/L (IQR: 1.6), *P* < 0.001], C-reactive protein [2.57 mg/L (IQR: 14.22) vs. 1.20 mg/L (IQR: 2.79), *P* = 0.039], D-dimer [0.71 mg/L (IQR:0.9) vs. 0.38 mg/L (IQR: 0.5), *P* = 0.002], blood urea nitrogen [5.09 mmol/L (IQR: 3.44) vs. 4.09 mmol/L (IQR: 1.64), *P* = 0.019], creatine kinase isoenzymes [11.58 ± (SD) 11.744 IU/L vs. 9.27 ± (SD) 8.55 IU/L, *P* = 0.007], and lactate dehydrogenase [194.78 ± (SD) 55.18 IU/L vs. 175.26 ± (SD)54.35 IU/L, *P* < 0.001] were much higher in patients with CVD. Whereas, lymphocyte count [1.11 ± (SD) 0.50 10^9^/L vs. 1.45 ± (SD) 0.64 10^9^/L, *P* < 0.001] and albumin [33.90 g/L (IQR: 7.75) vs. 37.20 g/L (IQR: 5.7), *P* < 0.001] were lower in patients with CVD.

**Table 2:** Laboratory examination of COVID-19 patients with or without history of CVD.

**Laboratory examination**	**History of CVD *n* = 109**	**No history of CVD *n* = 2,445**	***P*-Value**
White blood cell count (10^9^/L), median (IQR)	5.80 (5.85)	5.40 (2.3)	0.001
Neutrophil count (10^9^/L), median (IQR)	3.73 (3.02)	3.13 (1.6)	<0.001
Lymphocyte count (10^9^/L), mean ± SD	1.11 ± 0.50	1.45 ± 0.64	<0.001
C-reactive protein (mg/L), median (IQR)	2.57 (14.22)	1.20 (2.79)	0.039
Fibrinogen (g/L), mean ± SD	2.84 ± 0.74	2.93 ± 0.80	0.288
Thrombin time (S), mean ± SD	15.09 ± 1.11	15.08 ± 1.33	0.954
Prothrombin time (S), median (IQR)	12.99 (1.94)	12.65 (1.25)	0.756
Activated partial thromboplastin time (S), median (IQR)	27.48 (4.62)	27.62 (3.91)	0.634
D-dimer (mg/L), median (IQR)	0.71 (0.9)	0.38 (0.5)	0.002
Glutamic pyruvic transaminase (IU/L), mean ± SD	25.22 ± 22.59	31.04 ± 33.61	0.074
Glutamic oxaloacetic transaminase (IU/L), mean ± SD	24.19 ± 20.85	22.20 ± 24.42	0.402
Albumin (g/L), median (IQR)	33.90 (7.75)	37.20 (5.7)	<0.001
Blood urea nitrogen (mmol/L), median (IQR)	5.09 (3.44)	4.09 (1.64)	0.019
Serum creatinine (umol/L), median (IQR)	67.20 (41.60)	62.30 (20)	0.425
Creatine kinase (IU/L), median (IQR)	45.70 (70.78)	43.10 (34.8)	0.507
Creatine kinase isoenzymes (IU/L), mean ± SD	11.58 ± 11.74	9.27 ± 8.55	0.007
Lactate dehydrogenase (IU/L), mean ± SD	194.78 ± 55.18	175.26 ± 54.35	<0.001

*Data are reported as mean ± SD or median (IQR). T-test or Mann–Whitney U-test was used to calculate P-value when appropriate. Data of the following indicators were missing C-reactive protein in 1 case, Fibrinogen in 10 cases, Prothrombin time in 10 cases, Activated partial thromboplastin time in 10 cases, D-dimer in 10 cases, Creatine kinase in 1 case, Creatine kinase isoenzymes in 1case, Lactate dehydrogenase in 1case*.

During hospitalization, patients with a history of CVD were more likely to receive antibiotics (49.5 vs. 32.7%, *P* < 0.001), immune support (48.6 vs. 32.8%, *P* = 0.001), and glucocorticoids (23.9 vs. 14.1%, *P* = 0.005) than those without CVD. Besides, patients with COVID-19 and with CVD history had more severe clinical course. The proportions of mechanical ventilation (8.3 vs. 1.5%, *P* < 0.001), shock (8.3 vs. 1.9%, *P* < 0.001), and intensive care unit (ICU) admission (3.7 vs. 0.7%, *P* = 0.005) were much higher in the CVD group (Table 3).

**Table 3:** Clinical features and outcomes of COVID-19 patients with or without a history of CVD.

**Clinical features and outcomes**	**History of CVD *n* = 109**	**No history of CVD *n* = 2,445**	***P*-Value**
Time from symptoms to admission, mean ± SD	23.23 ± 14.59	24.07 ± 12.97	0.501
**Severity on admission (proportion)**			<0.001*
Mild cases	1 (0.9)	27 (1.1)	
Moderate cases	55 (50.5)	1,731 (70.8)	
Severe cases	49 (45.0)	660 (27.0)	
Critical cases	4 (3.7)	27 (1.1)	
**Drug treatment (proportion)**			
Antibiotics	54 (49.5)	799 (32.7)	<0.001
Antivirals	48 (44.0)	1,191 (48.8)	0.333
Immune support	53 (48.6)	801 (32.8)	0.001
Glucocorticoids	26 (23.9)	345 (14.1)	0.005
Mechanical ventilation (proportion)	9 (8.3)	36 (1.5)	<0.001
Non-Invasive	7 (6.4)	32 (1.3)	<0.001
Invasive	5 (4.6)	12 (0.5)	<0.001
ECMO	0	2 (0.1)	1.000
ICU care (proportion)	4 (3.7)	17 (0.7)	0.005
Shock (proportion)	9 (8.3)	47 (1.9)	<0.001
Length of hospital day, mean±SD	14.9 (9.9)	14.7 (8.7)	0.818
**Clinical outcomes (proportion)**			
Discharged	102 (93.6)	2,407 (98.4)	0.001
Died	7 (6.4)	38 (1.6)	0.001
Severe events (proportion)	14 (12.8%)	63 (2.6%)	<0.001

*Data are described as mean ± SD or proportion (%); χ^2^ test, Fisher exact test, T-test was used to calculate P-values when appropriate*.

### Patients With COVID-19 and With CVD History Have Worse Clinical Outcomes Compared to Patients With COVID-19 but Without CVD History

**Figure 1:**
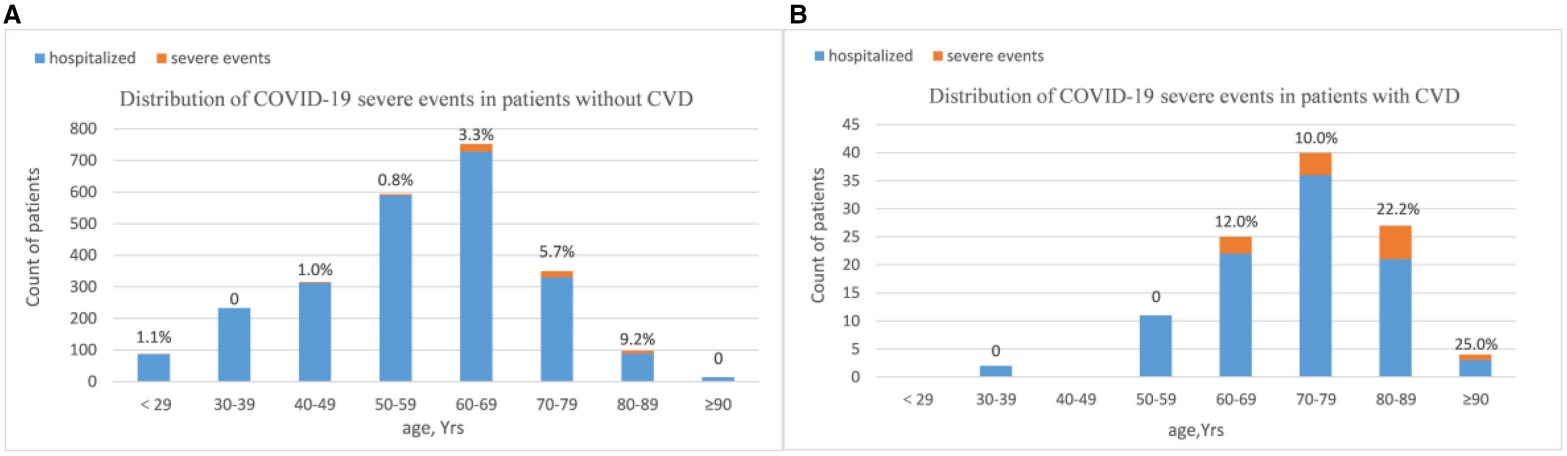
Distribution of COVID-19 severe events by CVD history [**(A)**: non-CVD subset and **(B)** CVD subset]. Bar plots show the counts of all hospitalized patients. Patients were stratified into age groups by decade. Counts (top of bars) show the percentage of patients who had severe events in respective age groups. The majority of severe events happened in patients over age sixty years. Among patients over sixty, the proportions of having severe events were much higher in CVD subset. Age distribution of patients with CVD history was right-skewed compared to the non-CVD subset.

Compared to patients without CVD, there was a lower rate of discharge (93.6 vs. 98.4%, *P* = 0.001) and a higher rate of mortality (6.4 vs. 1.6%, *P* = 0.001) in patients who had CVD. The percentage of patients with severe events comprised all-cause death, intensive care unit admission, shock, or mechanical ventilation usage during hospitalization was much higher in the CVD group (12.8 vs. 2.6, *P* < 0.01; Table 3). As shown in Figure 1, the age distribution of patients with CVD was right-skewed compared to the non-CVD subset. Most severe events occurred in patients over age 60 years. Among all the age groups over sixty, the proportion of critically ill patients were much higher in the CVD subset (age 60–69:12.0 vs. 3.3%; age 70–79:10.0 vs. 5.7%; age 80–89:22.2 vs. 9.2%; and age ≥90:25.0 vs. 0%). To minimize selection bias of age distribution, 109 patients with CVD history were matched to 545 patients without CVD in a 1:5 ratio by propensity score matching (PSM). After matching, there was an even distribution of age-level between groups (Supplementary Figure 1). The proportion of severe events was still higher in the CVD group (12.8 vs. 2.6%, *P* < 0.001).

Besides, we used logistic regression models to analyze the relationship between the CVD history and the severe events in the cohort of 2,554 cases. After adjusting for the confounding effects of age, sex, smoking, and comorbidities, the odds ratio for developing severe events with a history of CVD was 2.326 (95% CI, 1.168–4.630; *P* = 0.016).

### Risk Factors for Developing Severe Events in Patients With COVID-19 and With History of CVD

We have demonstrated that CVD patients were more likely to have severe events in the course of COVID-19 compared to patients without CVD history. Next, we try to figure out the risk factors for developing severe events in patients with CVD history. There were 109 patients with a medical history of CVD involved in our study cohort, including 97 cases of cerebral ischemia stroke, six cases of cerebral hemorrhage stroke, three cases of unspecified stroke, and three cases of severe cerebrovascular stenosis. There was no statistical difference between the CVD types and severe events (data did not show, *P* = 1.000). Variables that may be related to the clinical outcome, including baseline characteristics (age, sex, smoking history, and comorbidities), laboratory examinations, time from symptoms to admission, and drug treatments were included in the univariate regression analysis. As presented in Table 4, univariate analyses showed that increase in thrombin time, blood urea nitrogen, serum creatinine, creatine kinase, creatine kinase isoenzymes, lactate dehydrogenase, and decrease in lymphocyte count were significantly associated with severe events *(P* < 0.05). Multivariate analysis identified a decreased lymphocyte count (OR 9.192, 95% CI, 1.410–59.902, *P* = 0.020) and an increased blood urea nitrogen (OR 5.916, 95% CI, 1.072–32.641, *P* = 0.041) as the independent risk factors for developing severe events.

**Table 4:** Results of univariate and multivariate regression analysis of risk factors associated with severe events in COVID-19 patients with a history of CVD.

	**Univariate analysis**			**Multivariate analysis**		
**Variable**	**OR**	**95% CI**	***p*-value**	**OR**	**95% CI**	***p*-value**
Decreased lymphocyte count	6.286	1.641–24.076	0.007	9.192	1.410–59.902	0.020
Increased thrombin time	15.455	1.293–184.778	0.031	1.714	0.048–60.679	0.767
Increased blood urea nitrogen	4.937	1.521–16.027	0.008	5.916	1.072–32.641	0.041
Increased serum creatinine	3.944	1.242–12.523	0.020	2.120	0.414–10.853	0.367
Increased creatine kinase	6.905	1.813–26.295	0.005	2.809	0.373–21.131	0.316
Increased Creatine kinase isoenzymes	25.556	4.323–151.082	<0.001	5.601	0.547–57.370	0.147
Increased lactate dehydrogenase.	6.900	1.984–24.000	0.002	2.435	0.442–13.409	0.307

## Discussion

This study investigated the clinical characteristics, outcomes, and prognostic factors of patients with COVID-19 and with CVD history in a large cohort of 2,554 patients. Our result showed that after SARS-CoV2 infection, the risk of developing severe events was significantly higher in patients with CVD history than patients without. This result was consistent with several previous studies. Qin et al. demonstrated that patients with COVID-19 and with history of stroke were more likely to be admitted to the ICU, use mechanical ventilation, or die, compared to COVID-19 patients without a stroke history (7). Kummer et al. found that patients with a history of stroke had higher in-hospital mortality (8). However, data from other studies cast some doubt on this conclusion. A pooled analysis failed to find the statistical association between severe COVID-19 and the history of stroke (9). Besides, a retrospective study in China found that the comorbidities of COPD, diabetes, hypertension, and malignancy were the risk factors of severe COVID-19. However, a similar result was not found in the relationship between comorbidity of CVD and COVID-19 severity (4). There were only 19 and 30 patients with previous CVD included in each cohort, respectively. It cannot be ruled out that the small sample size may limit the reliability of these conclusions. A total of 2,554 cases were included in our study, including 109 cases with a history of CVD. Besides, the data analyzed in our study originated from an emergency specialty field hospital in Wuhan. A sufficient supply of medical materials and high levels of medical care were given guarantees by government priority policy, which provided relatively valid and reliable clinical data in the circumstances of emerging pandemic disease.

Stroke could induce the hyper-activation of the sympathetic nervous and cause a prolonged elevation of circulating catecholamines, subsequently leading to a decrease of peripheral immune cells and a type 2-biased helper T cell response at a systematic level (10, 11). As a result, patients who have a post-stroke are always related to immunosuppression status and are more susceptible to life-threatening infections. In our study, patients with CVD history were characterized with lower lymphocyte counts compared to patients without CVD. Besides, patients who had CVD with decreased lymphocyte counts were more likely to develop adverse outcomes, indicating that immunosuppression may be an important reason for suffering severe COVID-19 infection in patients with CVD history. Angiotensin-converting enzyme-2 (ACE2) has been established as a host receptor for SARS-CoV-2, which provides viral entry into human cells (12). There was an increased level of ACE2 in the pulmonary alveoli of stroke mice compared to sham-operated mice, suggesting the increased binding affinity of SARS-CoV-2, and a more aggressive inflammatory response in patients with previous brain injuries (13), which is another possible explanation of severe clinical course in patients with CVD history. Noticeably, the patients who had CVD in our study were generally older with more comorbidities. Consistent with this result, levels of creatine kinase isoenzymes and lactate dehydrogenase were higher, whereas the level of albumin was lower in the CVD patients, indicating more severe multiple organ dysfunction and worse nutritional status in those patients. Poor baseline well-being in patients who had CVD was also an essential interpretation of developing severe events in those participants. However, after adjusting the confounding effect of age distribution and comorbidities by propensity score matching and multivariate logistic regression analyses, the history of CVD was still independently associated with the incidence of severe events, suggesting that the brain injury itself has an important effect on the disease progression.

Apart from the decreased lymphocyte counts, our study found that the increased urea nitrogen level was another risk factor of disease severity in patients with CVD history. The main target of SARS-CoV-2 is the respiratory tract, causing initial respiratory symptoms (14, 15). However, ACE2 is also abundantly expressed in different cell types, including renal tubules (16). Extrapulmonary manifestations including renal failure (17, 18) in COVID-19 have been reported in patients with adverse outcomes. Our study suggested that the renal injury may play an important role in diseases deterioration in patients who had CVD. Early attention should be paid to these patients to prevent COVID-19 deterioration. Also, it is important to realize that the inherent limitations of retrospective studies made it difficult to determine whether there was a causal relationship between adverse outcomes and these hematologic biomarkers. More studies are still needed to get a more definite result.

Several limitations of our study should also be acknowledged. The information of comorbidities in this study was extracted from electronic medical records. Other underlying diseases associated with CVD, including hyperlipidemia, hyperuricemia, and hyperhomocysteinemia were not recorded in detail. Data on several variables such as stroke classification, level of neural function (NIHSS and mRS score), and home nursing quality were difficult to obtain in the circumstance of emerging infectious disease. Nevertheless, this study presented a whole picture of the clinical characteristics, outcomes, and risk factors of disease severity in patients with COVID-19 and with CVD history, which may provide clinicians with better management of patients who had CVD, who are prone to disease progression in SARS-CoV-2 infections.

## Data Availability Statement

The anonymized dataset will be made available on reasonable request to the corresponding author.

## Ethics Statement

The studies involving human participants were reviewed and approved by Ethics Committee of Guangdong Provincial Hospital of Traditional Chinese Medicine. The Ethics Committee waived the requirement of written informed consent for participation.

## Author Contributions

ML, JY, QG, JL, BG, and WC collected the data. ML, ZH, and HW performed statistical analyses. ML and JG drafted the manuscript. JG and BD designed the study. JG acted as the guarantor for the paper. All authors have read and agree to publish the final manuscript.

## Funding

This work was supported by the National Natural Science Foundation of China (Grant No. 81974559), Key Research and Development Program of Guangdong Province (Grant No. 2020B1111100009), Guangdong Provincial Key Laboratory of TCM Research on Emergency (Grant No. 2017B030314176), and the Natural Science Foundation of Guangdong Province (Grant No. 2021A1515011480).

## Conflict of Interest

The authors declare that the research was conducted in the absence of any commercial or financial relationships that could be construed as a potential conflict of interest.

## Publisher's Note

All claims expressed in this article are solely those of the authors and do not necessarily represent those of their affiliated organizations, or those of the publisher, the editors and the reviewers. Any product that may be evaluated in this article, or claim that may be made by its manufacturer, is not guaranteed or endorsed by the publisher.

## References

[1] World Health Organization. Weekly Epidemiological Update−1 December 2020. (2020). Available online at: https://www.who.int/publications/m/item/weekly-epidemiological-update–1-december-2020 (accessed December 5, 2020).

[2] Kissler SM Tedijanto C Goldstein E Grad YH Lipsitch M. Projecting the transmission dynamics of SARS-CoV-2 through the postpandemic period. Science. (2020) 368:860–8. 10.1126/science.abb5793 32291278 PMC7164482

[3] Gallo Marin B Aghagoli G Lavine K Yang L Siff EJ Chiang SS. Predictors of COVID-19 severity: a literature review. Rev Med Virol. (2021) 31:1–10. 10.1002/rmv.2146 32845042 PMC7855377

[4] Guan WJ Liang WH Zhao Y Liang HR Chen ZS Li YM. Comorbidity and its impact on 1590 patients with COVID-19 in China: a nationwide analysis. Euro Respir J. (2020) 55:2000547. 10.1183/13993003.01227-2020 32217650 PMC7098485

[5] Grasselli G Greco M Zanella A Albano G Antonelli M Bellani G. Risk factors associated with mortality among patients with COVID-19 in intensive care units in Lombardy, Italy. JAMA Intern Med. (2020) 180:1345–55. 10.1001/jamainternmed.2020.3539 32667669 PMC7364371

[6] Ho D Imai K King G Stuart EA. MatchIt: Nonparametric preprocessing for parametric causal inference. J Stat Soft. (2011) 42:1–28. 10.18637/jss.v042.i08

[7] Qin C Zhou L Hu Z Yang S Zhang S Chen M. Clinical characteristics and outcomes of COVID-19 patients with a history of stroke in Wuhan, China. Stroke. (2020) 51:2219–23. 10.1161/STROKEAHA.120.030365 32466735 PMC7282412

[8] Kummer BR Klang E Stein LK Dhamoon MS Jetté N. History of stroke is independently associated with in-hospital death in patients with COVID-19. Stroke. (2020) 51:3112–4. 10.1161/STROKEAHA.120.030685 32772679 PMC7467043

[9] Aggarwal G Lippi G Michael Henry B. Cerebrovascular disease is associated with an increased disease severity in patients with Coronavirus disease 2019 (COVID-19): a pooled analysis of published literature. Int J Stroke. (2020) 15:385–9. 10.1177/1747493020921664 32310015

[10] Shim R Wong CHY. Complex interplay of multiple biological systems that contribute to post-stroke infections. Brain Behav Immun. (2018) 70:10–20. 10.1016/j.bbi.2018.03.019 29571897

[11] Shi K Wood K Shi FD Wang X Liu Q. Stroke-induced immunosuppression and poststroke infection. Stroke Vasc Neurol. (2018) 3:34–41. 10.1136/svn-2017-000123 29600006 PMC5870641

[12] Hamming I Timens W Bulthuis ML Lely AT Navis G van Goor H. Tissue distribution of ACE2 protein, the functional receptor for SARS coronavirus. A first step in understanding SARS pathogenesis. J Pathol. (2004) 203:631–7. 10.1002/path.1570 15141377 PMC7167720

[13] Singh V Beer A Kraus A Mang F Zhang X Xue J. Stroke increases the expression of ACE2, the SARS-CoV-2 binding receptor, in murine lungs. Brain Behav Immun. (2021) 2021:458–62. 10.1016/j.bbi.2021.01.039 33621620 PMC7896496

[14] Guan WJ Ni ZY Hu Y Liang WH Ou CQ He JX. Clinical characteristics of coronavirus disease 2019 in China. N Engl J Med. (2020) 382:1708–20. 10.1056/NEJMoa2002032 32109013 PMC7092819

[15] Wang D Hu B Hu C Zhu F Liu X Zhang J. Clinical characteristics of 138 hospitalized patients with 2019 novel coronavirus-infected pneumonia in Wuhan, China. JAMA. (2020) 323:1061–9. 10.1001/jama.2020.1585 32031570 PMC7042881

[16] Hikmet F Méar L Edvinsson Å Micke P Uhlén M Lindskog C. The protein expression profile of ACE2 in human tissues. Mol Syst Biol. (2020) 16:e9610. 10.15252/msb.20209610 32715618 PMC7383091

[17] Cheng Y Luo R Wang K Zhang M Wang Z Dong L. Kidney disease is associated with in-hospital death of patients with COVID-19. Kidney Int. (2020) 97:829–38. 10.1016/j.kint.2020.03.005 32247631 PMC7110296

[18] Ok F Erdogan O Durmus E Carkci S Canik A. Predictive values of blood urea nitrogen/creatinine ratio and other routine blood parameters on disease severity and survival of COVID-19 patients. J Med Virol. (2021) 93:786–93. 10.1002/jmv.26300 32662893 PMC7405288

