# Supplementary material for: Clinical Characteristics, Outcomes, and Risk Factors of Disease Severity in Patients With COVID-19 and With a History of Cerebrovascular Disease in Wuhan, China: A Retrospective Study
Source: Front Neurol. 2022 Jan 11;12:706478. doi: 10.3389/fneur.2021.706478 (PMC8787151; doi:10.3389/fneur.2021.706478)
Supplement: Supplementary file 1 [file Image_1.pdf]

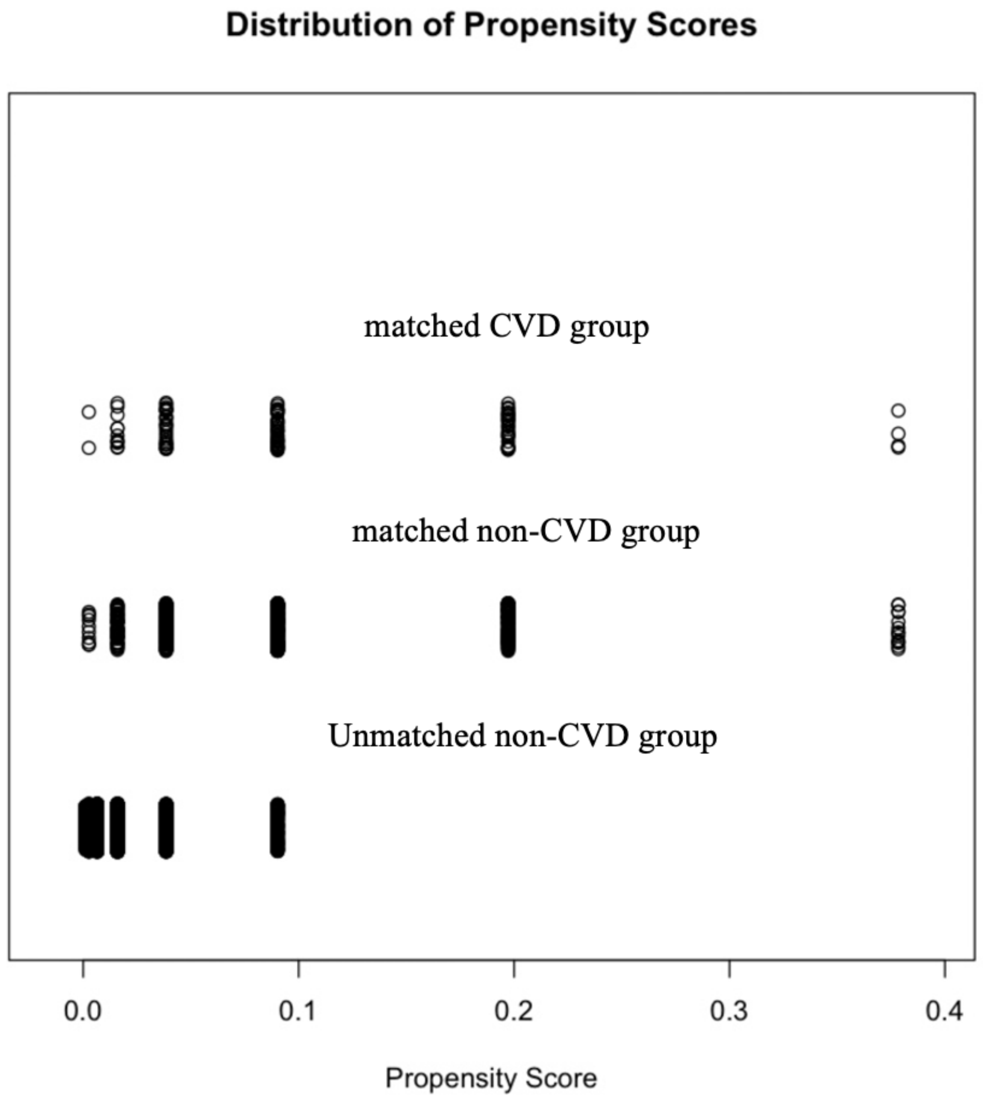


Supplement Figure: 1:5 propensity score matching analysis (109 CVD patients: 545 non-CVD patients) was conducted by 'MatchIt' package in R software. After matching, there is an even distribution of age-level between groups
